# Metastasis-Inducing S100A4 and RANTES Cooperate in Promoting Tumor Progression in Mice

**DOI:** 10.1371/journal.pone.0010374

**Published:** 2010-04-28

**Authors:** Birgitte Forst, Matilde Thye Hansen, Jörg Klingelhöfer, Henrik Devitt Møller, Gitte Helle Nielsen, Birgitte Grum-Schwensen, Noona Ambartsumian, Eugene Lukanidin, Mariam Grigorian

**Affiliations:** Department for Molecular Cancer Biology, Danish Cancer Society, Copenhagen, Denmark; University of Illinois at Chicago, United States of America

## Abstract

**Background:**

The tumor microenvironment has been described as a critical milieu determining tumor growth and metastases. A pivotal role of metastasis-inducing S100A4 in the development of tumor stroma has been proven in animal models and verified in human breast cancer biopsies. Expression and release of S100A4 has been shown in various types of stroma composing cells, including fibroblasts and immune cells. However, the events implicated in upstream and downstream pathways regulating the activity of the extracellular S100A4 protein in the tumor milieu remain unsolved.

**Methodology/Principal Findings:**

We studied the interplay between the tumor cell-derived cytokine **r**egulated-upon-**a**ctivation, **n**ormal **T**-cell expressed and **s**ecreted (RANTES; CCL5) and S100A4 which were shown to be critical factors in tumor progression. We found that RANTES stimulates the externalization of S100A4 via microparticle shedding from the plasma membrane of tumor and stroma cells. Conversely, the released S100A4 protein induces the upregulation of fibronectin (FN) in fibroblasts and a number of cytokines, including RANTES in tumor cells as well as stimulates cell motility in a wound healing assay. Importantly, using wild type and S100A4-deficient mouse models, we demonstrated a substantial influence of tumor cell-derived RANTES on S100A4 release into blood circulation which ultimately increases the metastatic burden in mice.

**Conclusions/Significance:**

Altogether, the data presented strongly validate the pro-metastatic function of S100A4 in the tumor microenvironment and define how the tumor cell-derived cytokine RANTES acts as a critical regulator of S100A4-dependent tumor cell dissemination. Additionally, for the first time we demonstrated the mechanism of S100A4 release associated with plasma membrane microparticle shedding from various cells types.

## Introduction

Over the past decade, the intriguing model of tumor development emerged based on the concept that throughout the entire process of cancer etiology, progression, and metastasis, the tumor microenvironment could be an active participant. The cross-talk between tumor and stroma cells could be mediated through direct heterotypic cell-cell contacts or secreted molecules comprising growth factors, cytokines and extracellular matrix proteins. The production and release of these factors implicate both tumor and various types of physiologically altered stroma cells, such as fibroblasts and immune and vasculature composing cells [1]–[3].

S100A4, a small Ca^2+^-binding protein of the S100 family, is an essential pro-metastatic mediator in tumor and is categorized as a useful prognostic marker in numerous tumor types [4], [5]. Moreover, S100A4-deficient mice exhibit delayed tumor uptake, impaired stroma organization and suppression of metastasis [4], [6]–[9]. S100A4 binds to several intracellular target proteins (e.g., p53, non-muscle myosin-IIA, liprin β1 and others) and modulates gene expression, cell motility and adhesion [10]–[14]. Self-aggregation of S100A4 produces extracellularly active forms of the protein [15].

Secretion of S100A4 from tumor and stroma cells was demonstrated *in vitro* and elevated S100A4 protein levels were detected in blood of S100A4 transgenic mice [9], [6]. As an extracellularly active protein, S100A4 stimulates angiogenesis [16], upregulates matrix metalloproteinases (MMPs), downregulates tissue inhibitors of MMPs (TIMPs) in endothelial and tumor cells [6], [7], [17], [18], promotes neurite outgrowth and survival of primary hippocampal cells [15], [19], and promotes migration of astrocytic tumor cells [20]. The functional significance of extracellular S100A4 was also shown in periodontal ligaments [21] and cardiomyocyte differentiation and hypertrophy [22]. Moreover, our recent data demonstrated strong upregulation of S100A4 in various cell types (e.g. fibroblasts and immune cells), not only in tumor stroma [9], but also in synovial tissue of rheumatoid arthritis patients [23] and involved skin dermis of patients with psoriasis. Importantly, anti-S100A4 antibodies inhibited the pathological symptoms of psoriasis in a mouse model [24]. Altogether, these observations indicate an important extracellular role of S100A4 *in vivo* and suggest a putative active role of S100A4 in the tumor milieu, most likely in its proinflammatory pathway(s).

We explored the factors and pathways implicated in S100A4 activation in the tumor microenvironment. We previously demonstrated the induction of S100A4 release from fibroblasts mediated by conditioned media (CM) from VMR (metastatic), but not CSML0 (non-metastatic) cells [7]. In the present study we identified the tumor cell-derived cytokine **r**egulated-upon-**a**ctivation, **n**ormal **T**-cell expressed and **s**ecreted RANTES (CCL5) as a strong inducer of S100A4 release from various cell types and determined that RANTES-mediated cytoskeleton-associated shedding of microparticles is a main route of S100A4 externalization. We also demonstrated feedback effects of extracellular S100A4 on tumor and stroma cells, including activation of cytokines and RANTES in particular. Furthermore, we showed that the S100A4/RANTES interplay significantly promotes metastatic features of tumor cells.

## Results

### Identification of cytokines specific for metastatic *vs* nonmetastatic tumor cells

We previously suggested the existence of soluble tumor cell-derived factor(s) which induces the stimulation of S100A4 externalization from different tumor stroma cells [7]. To characterize these factors, we performed a differential screening with CM from metastatic mouse adenocarcinoma cell line VMR revealing stimulatory effect on S100A4 release from fibroblasts and inactive CM from nonmetastatic mouse adenocarcinoma CSML0. CM were harvested from cells grown for 24 h and probed using mouse cytokine antibody arrays.

**Figure 1:**
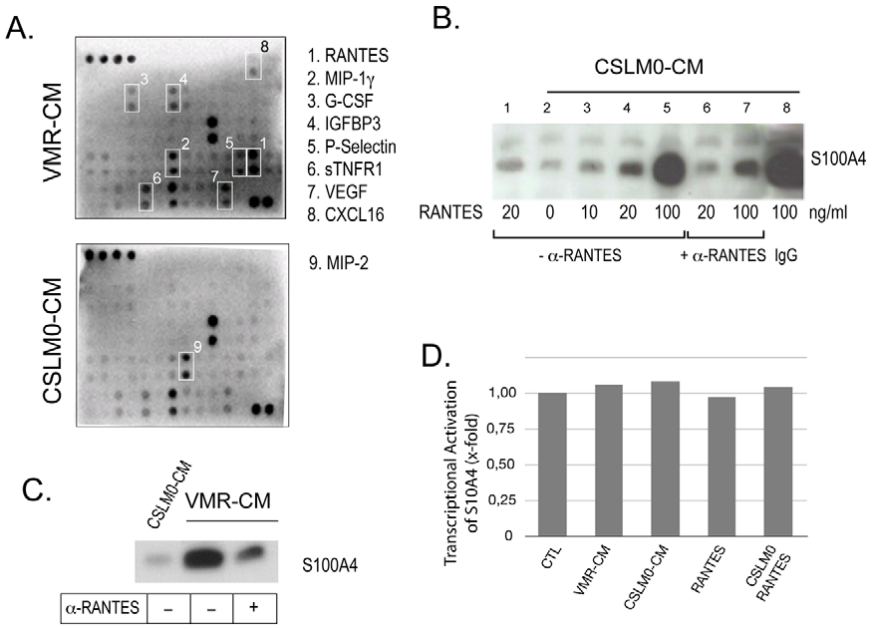
RANTES-mediated induction of S100A4 release from 4MEF. (**A**) Differential screening of VMR-CM and CSML0-CM by a cytokine antibody array. Upregulated cytokines are marked with white rectangles. (**B**) Western blot analysis of S100A4 released into CM in response to increasing concentrations of recombinant RANTES added to CSML0-CM (lane 2–8) and the inhibitory effect of rabbit anti-RANTES antibodies on RANTES-mediated S100A4 release (lane 6–7). Rabbit IgG was used as a negative control (lane 8). (**C**) Western blot analysis of S100A4 in CM of 4MEF in response to VMR-CM and anti-RANTES antibodies. (**D**) A representative experiment (qPCR) demonstrating lack of S100A4 transcriptional activation in 4MEF in response to various treatments.

The array analyses generally revealed significantly higher complexity and levels of cytokines in VMR-CM compared with CSML0-CM. We found RANTES/CCL5, MIP-1γ/CCL9, VEGF, p-Selectin, IGFBP3, IGFBP-5, sTNFR1, G-CSF, and CXCL-16 as the most prominently expressed VMR-specific cytokines (Fig. 1A), whereas MIP-2/CXCL-2 appeared to be the only cytokine differentially expressed in CSML0 and was not detected in VMR-CM (Fig. 1A). We selected RANTES for further studies because it was the highest upregulated cytokine in VMR-CM and its documented implication in tumor progression [25], [26].

First, we studied whether recombinant RANTES can stimulate S100A4 secretion from fibroblasts. We found that RANTES did not reveal any stimulatory activity when added directly to cell culture media (DMEM/10% FCS). However, when we supplemented recombinant RANTES with CSML0-CM, we observed S100A4 secretion from fibroblasts in a dose dependent manner, suggesting that a certain factor(s) in CSML0-CM cooperatively act with RANTES (Fig. 1B). Additionally, we showed that S100A4 release induced by both VMR-CM and recombinant RANTES could successfully be circumvented by anti-RANTES, but not by control IgG which substantiates the involvement of RANTES in this process (Fig. 1B and C). We next demonstrated by means of quantitative PCR (qPCR) analyses that enhanced S100A4 release from fibroblasts was not preconditioned by its transcriptional activation (Fig. 1D). These results clearly showed a RANTES-driven activation of S100A4 release from cultured fibroblasts.

### Mechanisms of RANTES-mediated S100A4 externalization

**Figure 2:**
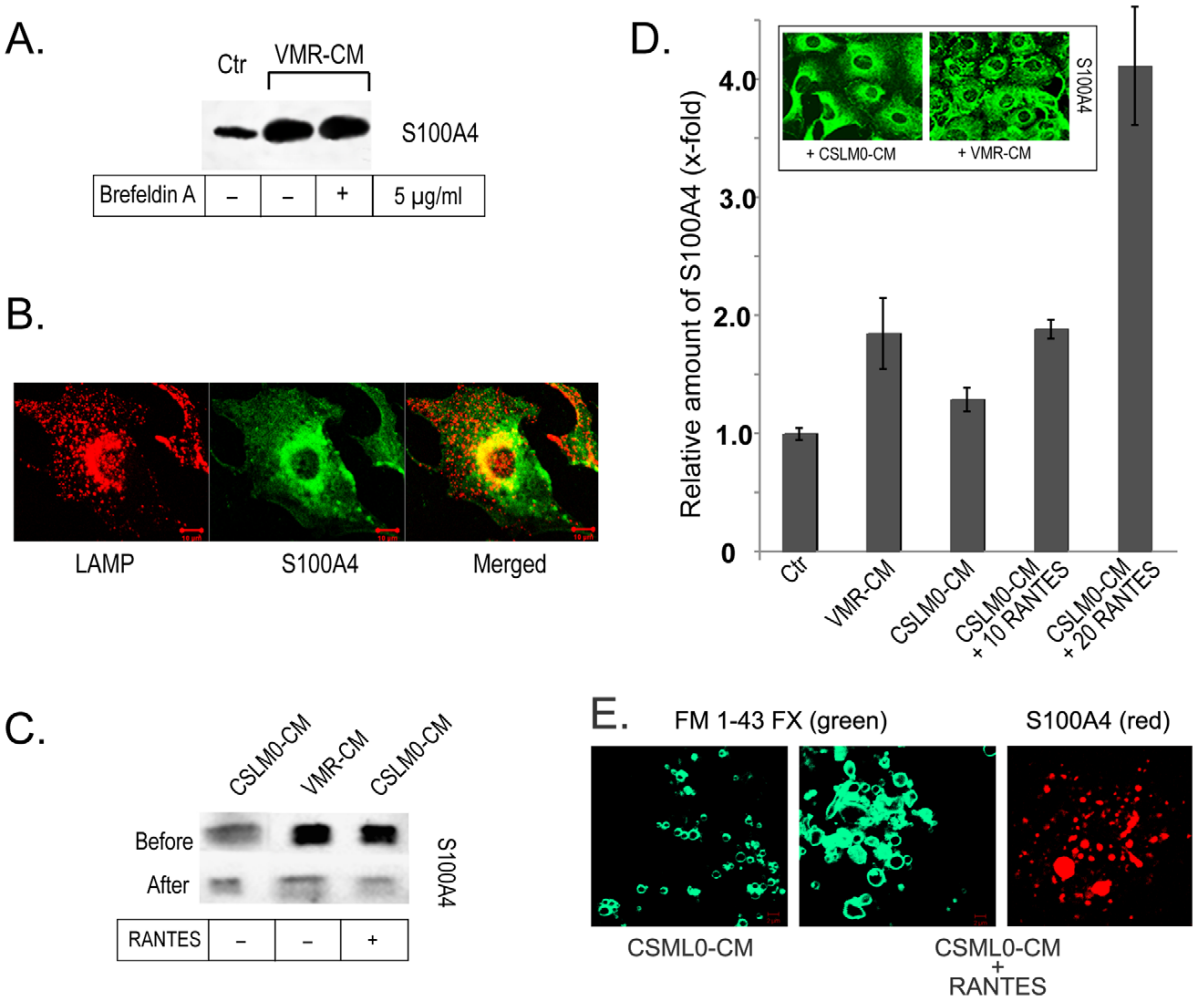
Mechanism of S100A4 externalization. (**A**) Western blot analysis of S100A4 in 4MEF CM. Brefeldin A did not affect S100A4 secretion. (**B**) Double immunofluorescence of 4MEF with anti-S100A4 and anti-LAMP1 (lysosomal marker) antibodies. (**C**) Western blot of S100A4 in CM from stimulated 4MEF before and after microparticle depletion. (**D**) Sandwich ELISA of S100A4 in microparticles released from 4MEF in response to VMR-CM, CSML0-CM, 10 and 20 ng/ml RANTES in CSML0-CM. (Inset) Appearance of S100A4-positive microparticle-like structures in fibroblasts stimulated with VMR-CM. (**E**) Immunofluorescence analysis of macroparticle-containing fraction (100K pellet) from CM of cells treated with CSML0-CM and CSML0-CM+RANTES labeled with lipophilic dye FM®1–43FX (live, green) and anti-S100A4 antibodies (fixed, red).

Given the critical extracellular role of S100A4 during tumor progression, we explored the mechanisms responsible for the active release of S100A4 in the tumor microenvironment. We found that the conventional ER/Golgi secretory pathway is not implicated in S100A4 release because the inhibition of this pathway by Brefeldin A did not interfere with VMR-CM-stimulated S100A4 externalization from fibroblasts (Fig. 2A).

VMR-CM-stimulated fibroblasts were then analyzed by immunofluorescence microscopy using antibodies against the lysosomal marker LAMP-1 and S100A4 (Fig. 2B). However, we were not able to demonstrate localization of LAMP-1 and S100A4 in externalized lysosomes, which rather excludes a role of the secretory lysosomal pathway in S100A4 release.

Next, we examined S100A4 externalization by microparticle shedding from the plasma membrane [27]. We fractionated CM from stimulated and control cells using sequential centrifugation and collected microparticles in the pellet at 100,000×*g* (100K) fraction [28], [29]. We showed that the depletion of microparticles by ultracentrifugation from the VMR-CM and RANTES/CSLM0-CM was accompanied by a substantial decrease of S100A4 in CM as analyzed by Western blotting (Fig. 2C). On the other hand a significant increase of the S100A4 protein in the pelleted microparticle fraction was measured by sandwich ELISA (Fig. 2D). Similar results were obtained with mouse monocyte/macrophage RAW 264.7, mammary adenocarcinoma CSML100 and human fibroblastic WI-38 cell lines (data not shown) indicating that suggested mechanism of S100A4 externalization is rather common.

To characterize the composition of the 100K pellet we stained the resuspended material with the Lipophilic dye FM® 1–43FX which is widely used to study the plasma membrane and vesiculation by immunofluorescence microscopy. We observed vesicle-like structures in the 100K pellet and their amount was clearly higher after stimulation with VMR-CM *vs* CSML0-CM (Fig. 2E). The increased amount of microvesicles correlated well with higher protein content in the pellets. Based on this observation we assessed in further experiments the S100A4 protein content in pellets to estimate the efficiency of microvesicle formation. Additionally, a smear of microvesicles from the same preparation showed round-shaped S100A4-positive structures indicating the localization of S100A4 in microvesicles (Fig. 2E, right panel).

**Figure 3:**
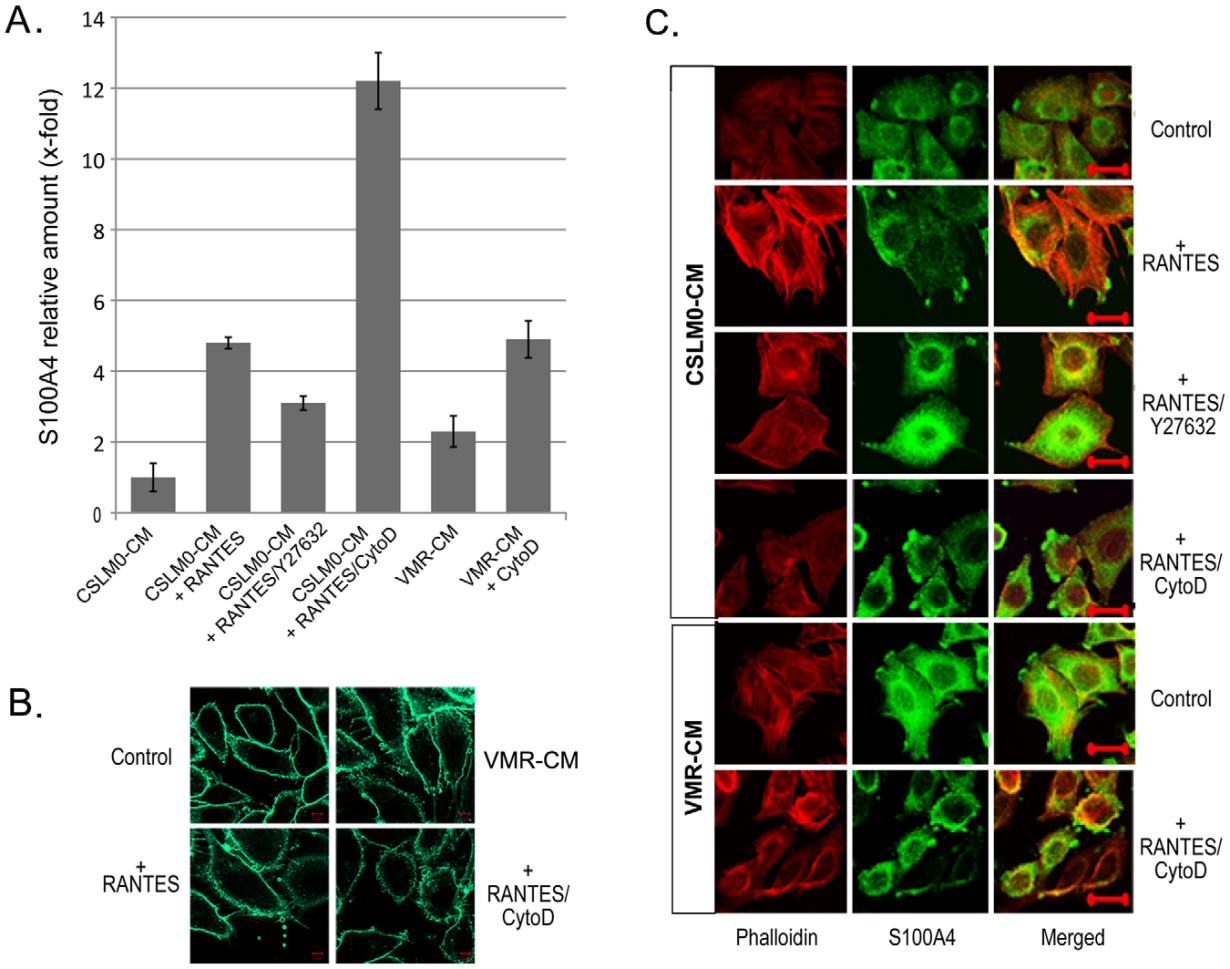
Cytoskeleton-associated transport of S100A4 in microparticles. (**A**) Sandwich ELISA of S100A4 in microparticles released from CSML100 cells treated with CSML0-CM, 20 ng/ml RANTES in CSML0-CM, 20 ng/ml RANTES in CSML0-CM+Y27632, 20 ng/ml RANTES in CSML0-CM+Cytochalasin D, VMR-CM, and VMR-CM+Cytochalasin D. (**B**) Immunofluorescence live imaging of plasma membrane structure of adherent CSML100 cells labeled with the lipophilic dye FM®-1–43FX by different treatments as indicated. (**C**) Cells after the same treatments were visualized with double-immunofluorescence with anti-S100A4 antibodies (green) and rhodamine phalloidin (red). Scale bar = 50 µM.

The actin cytoskeleton has been associated with microparticle formation earlier [27]. Therefore we explored its possible role in the S100A4 transport and release. For these experiments we used CSML100 tumor cells which have been shown to form large amounts of S100A4-carrying microvesicles upon stimulition. We used inhibitors that manipulate actin stress fibers, namely Y-27632 (10 µM; an inhibitor of Rho-associated protein kinase [ROCK]) and Cytocholasin D (200 nM; a potent inhibitor of actin polymerization). Surprisingly, we observed an inhibitory effect on the release of S100A4 by Y-27632 and a stimulatory effect on the release of S100A4 by Cytochalasin D in CSML100 cells using sandwich ELISA of the CM (Fig. 3A). More, labeling live cells with the Lipophilic dye FM®1–43FX revealed correlation between the levels of released S100A4 and the intensity of plasma membrane-derived microparticle staining (Fig. 3 B). We also observed a correlation between the level of released S100A4 and the amount of S100A4-positive vesicle-like structures determined by immunofluorescence of the fixed cells (Fig. 3 C). We observed that in CSML100 cells Cytochalasin D induced the depolymerization of actin stress fibers, while Y-27632 altered the stress fibers from cell traversing fibers into plasma membrane associated F-actin cortical fibers (Fig. 3C). The alterations in the architecture of actin fibers might explain the difference in the formation and release of microparticles.

In these experiments we carefully tested that the formation of microparticles is not associated the induction of apoptosis by examining along the LDH activity (data not shown) also activation of Caspase −3 and nuclear shrinkage (Supplementary Fig. S1).

### Functional impact of S100A4 carrying microparticles on tumor and stroma cells

**Figure 4:**
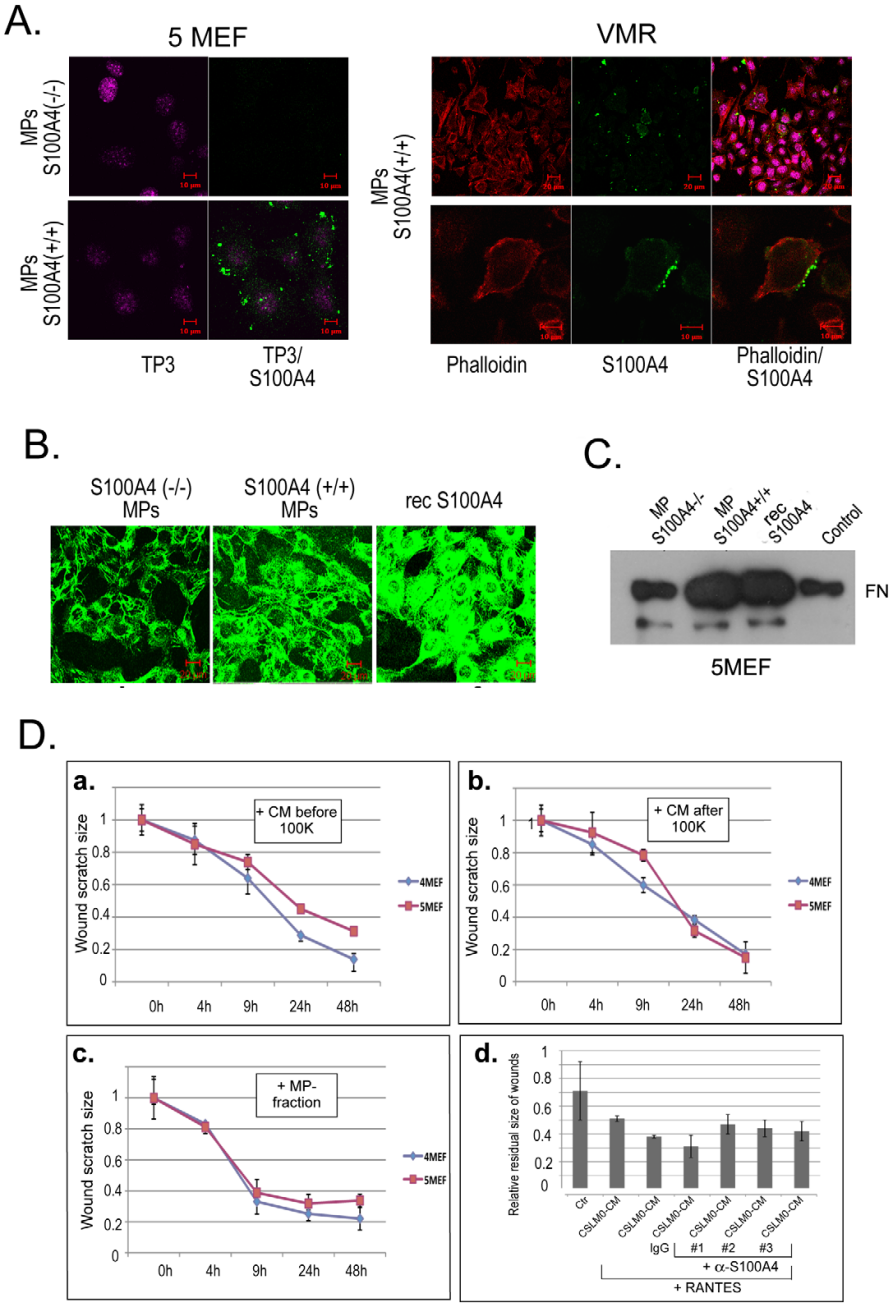
Functional significance of extracellular S100A4. (**A**) Distribution of microparticles isolated from S100A4-positive 4MEF cells added to 5MEF and VMR cells. Immunofluorescence staining was performed with anti-S100A4 antibodies (green), rhodamine phalloidin (red), and nuclear staining with TO-Pro (TP3) (pink). (**B**) Immunofluorescence detection of FN (green) in 5MEF cells in response to S100A4+/+ and S100A4−/− microparticles from 4MEF and 5MEF cells, and 1µg/ml of the recombinant oligomeric S100A4 protein, respectively. (**C**) Detection of FN by Western blot analysis of cell lysates from 5MEF treated with S100A4−/− and S100A4+/+ microparticles and recombinant S100A4. As a control cell lysate from non-treated cells were used. FN band corresponding to molecular weight of approximately 250 kDa is indicated. (**D**) Effects of S100A4 microparticles on wound healing in 5MEF cells. Conditioned media from 4MEF and 5MEF cells before (a) and after (b) 100,000×g centrifugation and isolated microparticles (c) were added to scratched monolayers of 5MEF cells. Time-course kinetics of residual wounds are depicted in the graphs. (d) Wound healing assay with 4MEF cells. The residual size of scratches 12 h after “healing” is presented. Three different batches (#1, 2 and 3) of affinity purified polyclonal anti-S100A4 antibodies were used.

To investigate the potential function of S100A4-containing MPs in the stroma/tumor cells crosstalk we studied the effect of fibroblast originated S100A4-containing MPs on tumor cells. First we monitored the distribution of the MPs after adding them to VMR and S100A4−/− 5MEF target cells. The experiments revealed that the added microparticles were not only distributed within the surrounding cellular spaces but also detected on the cell membrane and in the cytoplasm. Microparticles from S100A4−/− 5MEF cells served as negative control for the S100A4 immunofluorescence staining (Fig. 4A). We found that almost 100% of 5MEF cells and less than 10% of VMR cells were competent in microparticle uptake. This difference indicates an active mechanism in MP uptake involving cell surface molecules.

Earlier we obtained preliminary data indicating a S100A4-driven activation of FN. Therefore we analyzed whether S100A4 enriched in microparticles could stimulate FN production in fibroblasts. For that we tested microparticles derived from both S100A4+/+ and S100A4−/− fibroblasts. The 5MEF showed significantly stronger response in FN activation from S100A4-positive *vs* S100A4-negative microparticles, as determined by both, immunofluorescence staining (Fig. 4B) and Western blotting (Fig. 4C). Noteworthy, the recombinant S100A4 protein induced FN production in a similar way. These data clearly demonstrate a stimulatory effect for both, S100A4, enriched in microparticles and the recombinant S100A4 protein on FN production in fibroblasts.

Based on the documented influence of FN on cell migration [30], we examined the influence of S100A4 microparticles on cell motility in wound healing experiments. “Wounded” monolayer of 5MEF cells were treated with microparticle fractions as well as CM before and after microparticle depletion. We found a higher stimulatory effect with CM from S100A4+/+ 4MEF cells compared to CM from S100A4-deficient 5MEF on the wound healing speed (Fig. 4D-a). Moreover, depletion of CM from microparticles attenuated this difference (Fig. 4D-b), suggesting a role for S100A4-carrying microparticles in cell motility stimulation. Indeed, microparticles reconstituted from pellets after centrifugation revealed a small but reproducible effect on cell motility, in which S100A4+/+ microparticles stimulated 5MEF motility better than S100A4-/− (Fig. 4D-c). Removal of microparticles from CM did not influence the cell proliferation rate (data not shown), suggesting that the relative speed of wound healing is preconditioned by cell motility.

We next sought for the impact of extracellular S100A4 induced by RANTES on cell motility in a wound healing assay using S100A4-positive 4MEFs. We found that CSLM0-CM alone increased cell motility by 28%, whereas CSML0-CM supplemented with RANTES increased cell motility by 55%. Importantly, anti-S100A4 antibodies (#1, #2 and #3) but not rabbit IgG blocked this effect (Fig. 4D-d). This observation indicates that RANTES-induced acceleration of cell motility is at least partially mediated by the S100A4 release.

**Figure 5:**
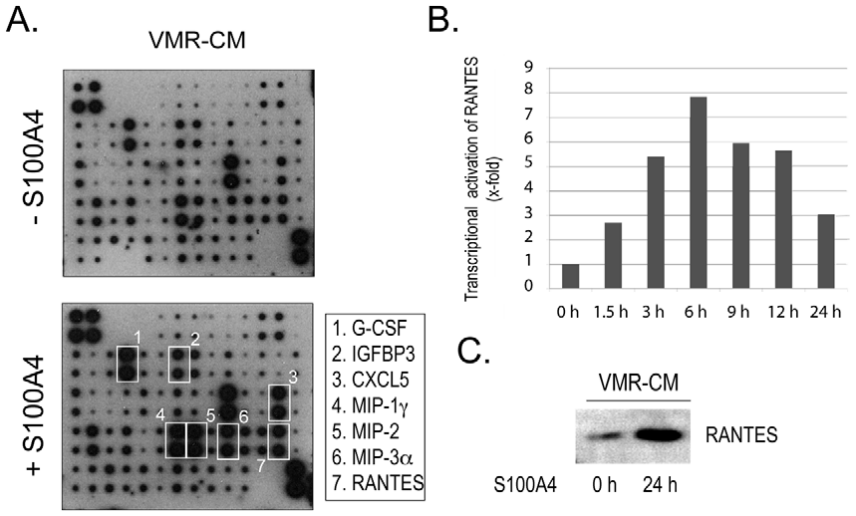
S100A4-mediated activation of cytokines in VMR cells. (**A**) Cytokine antibody array analysis of CM from VMR cells, unstimulated and stimulated with 0.5 µg/ml recombinant oligomeric S100A4. Upregulated cytokines are marked with white rectangles. (**B**) Kinetics of RANTES transactivation in response to 0.5 µg/ml recombinant oligomeric S100A4 (qPCR). (**C**) Western blot analysis of S100A4-mediated upregulation of RANTES in VMR-CM.

Furthermore, we analyzed the content and level of cytokines in CM from VMR cells responded to treatment with active oligomeric S100A4. Data obtained by the cytokine antibody array revealed an upregulation of several cytokines (e.g. G-CSF, RANTES and more) in S100A4-treated compared with non-treated VMR cells (Fig. 5A). The upregulation of RANTES was verified by using quantitative real-time PCR (qRT-PCR) and Western blot analysis. We observed both, a S100A4-mediated bell-shaped transcriptional activation of RANTES in VMR cells after treatment by S100A4, with a peak at 6 h (Fig. 5B) and an increased level of RANTES in the CM at 24 h (Fig. 5C).

### Rantes/S100A4 crosstalk promotes lung metastases

To investigate the significance of the RANTES/S100A4 pathway in tumor development and metastases, we generated RANTES-expressing VMR and CSML0 cell lines and the corresponding mock-infected counterparts. To characterize their tumorigenic properties we employed two different experimental setups where VMR-mock and VMR-RANTES cells were injected intravenously into syngeneic mice followed by monitoring animal survival and metastasogenesis. To assess the survival rate, mice were sacrificed at the time-points of the maximal morbidity defined by Danish Law on Animal Experimentation. We found that enhanced RANTES expression in VMR-RANTES vs the control group (Supplementary Fig. S2) significantly reduced the animal survival rate (*p = 0.0021*) with an average survival of 34.5 days and 46 days, respectively (Table 1). Notably, in these experiments, mice injected with VMR-RANTES exhibited a wider spectrum of organs displaying metastasis. Thus, 80% of mice (5 of 6) developed metastasis in organs other than the lung and liver, such as bone, lymph node, intestine, ovary and kidney.

**Table 1:** Survival rate and metastases among animal groups of A/Sn wild type mice injected with VMR-vector and -RANTES cell lines.

Cell lines	NN of mice with metastases	Survival (average)	Metastases in lungs	Metastases in liver	Metastases in other organs
VMR-vector	4/6	**46** * (**±**4.9)	3/6	4/6	0/6
VMR-Rantes	5/6	**34** (**±**5.1)	6/6	5/6	5/6

***p = 0.0021.***

*Two mice were killed due to sufficient length of the experiment (more than 46 days) without obvious symptoms.

By the next experimental setup, we compared the metastatic burden (metastasogenesis) in the lung and liver of mice inoculated i/v by VMR cells overexpressing RANTES *vs* empty vector at fixed time-points. Experiments with VMR cell lines were carried out with two mouse strains, S100A4-null and wild type A/Sn. In wild type mice inoculated with VMR-RANTES cells, we monitored lesions in lungs (6/6), liver (6/6), and other organs (4/6), whereas wild type mice inoculated with VMR-mock cells displayed little or no metastasis at this time-point. In S100A4-null mice VMR-RANTES displayed a more moderate metastatic capacity with fewer metastatic lesions, especially in lungs (1/6). The difference in metastatic phenotype between VMR-RANTES and VMR-vector in S100A4-null mice was thus less pronounced than in wild type animals.

**Figure 6:**
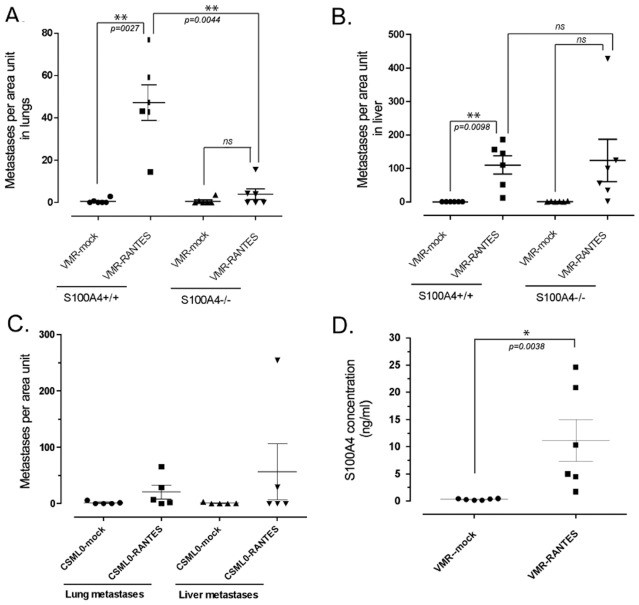
S100A4-associated RANTES-driven metastatatic capacity of tumor cells. (**A**) Metastatic burden indicated as a number of lesions per area unit in lungs of wild type (S100A4+/+) and S100A4−/− A/Sn mice inoculated i/v with VMR-mock and -RANTES cells. n = 6 per group. (**B**) Metastatic burden in liver in wild type (S100A4+/+) and S100A4−/− A/Sn mice inoculated i/v with VMR-mock and -RANTES cells. n = 6 per group. (**C**) Lung and liver metastatic burden in wild type A/Sn mice inoculated i/v with CSML0-mock and -RANTES cells. n = 5 per group, *p = 0.1060* (lung metastases) and *p = 0.7241*(liver metastases). (**D**) S100A4 concentration in the serum of A/Sn mice inoculated with VMR-mock and -RANTES cells determined by sandwich ELISA.

Statistical analysis of the metastatic burden (metastases per organ area unit) revealed significant influence of RANTES on the metastatic phenotype of tumor cells. Thus the metastatic burden in both lung and liver was substantially increased and statistically significant in wild type (S100A4+/+) mice inoculated with VMR-RANTES *vs* VMR-mock cells with a *p-value* of *0.0027* for lung metastases, and *0.0098* for liver metastases (Fig. 6 A and B). In the case of S100A4-deficient mice we observed a modest increase in lung (*p = 0.3261*) and more pronounced increase in liver metastases (*p = 0.1098*), though statistically insignificant. However a significant difference between metastatic burden in lungs in wild type and S100A4−/− mice (*p = 0.0044*) was the most striking observation in these experiments, suggesting an impact of RANTES/S100A4 circuit in stimulation of lung metastasis (Fig. 6A) in contrast to the liver metastases, where the difference in metastatic burden in the two mouse strains was negligible and insignificant (Fig. 6B).

Assessment of the metastatic burden in mice inoculated with cell lines originated from non-metastatic CSML0 adenocarcinoma cell line, (CSML0-RANTES vs CSML0-mock) confirmed the influence of RANTES in stimulation of metastases, although being statistically insignificant, *p = 0.2* for lung and *p = 0.32* for liver metastases (Fig. 6C).

To associate the effects of RANTES on the metastatic burden *in vivo* with the activation of S100A4 release, we analyzed S100A4 concentrations in mouse serum by sandwich ELISA. We observed detectable and statistically significant (*p = 0.038*) quantities of S100A4 (15±10 ng/ml) in the serum of mice inoculated with VMR-RANTES but not VMR-vector (Fig. 6D).

Altogether, the data verified the significance of RANTES/S100A4 alliance in the stimulation of the malignant properties of tumor cells and pointed its possible role in development of organospecific metastases.

## Discussion

Tumor progression and the process of metastasis formation involves tumor-stroma interactions, including a wide range of cellular (e.g. fibroblasts and immune and vascular cells) and molecular components (e.g. growth factors, cytokines, proteases and extracellular matrix proteins) which interact to build a solid foundation for tumor malignancy. Strong upregulation and release of the metastasis-inducing S100A4 protein was demonstrated in tumor stroma [9]. The prediction of factors in metastatic *vs.* non-metastatic tumor cells able to stimulate the release of S100A4 from fibroblasts and other tumor composing cells was made previously [7].

Data obtained on a remarkable difference in the repertoire and level of cytokines produced and secreted by metastatic cells (VMR) *vs.* non-metastatic cells (CSML0) was rather expected. Thus, cytokines reported as contributors to tumor progression, such as RANTES, MIP-1γ/CCL9, VEGF [31], p-Selectin [32], G-CSF [33], and CXCL-16 [34], are upregulated in VMR-CM, whereas only the metastasis inhibitor MIP-2 [35] is increased in CSML0-CM. The involvement of the most upregulated in VMR-CM cytokine RANTES has been reported in the progression of different tumor types, particularly in breast cancer [26], [36], [37]. RANTES was originally identified as a leukocyte chemoattractant factor [38], which is expressed by tumor cells and acts as a strong chemoattractant contributing to tumor progression [39]. However, the precise involvement of RANTES in malignancy remains unknown. We suggest a mechanism in which RANTES, by mediating S100A4 release from stroma cells into the tumor milieu, by a mechanism not affecting the *S100A4* gene expression enhances the metastatic capability of tumor cells. Furthermore, we suggest that RANTES-mediated S100A4 secretion induces the upregulation of cytokines (including RANTES), the production of FN, the stimulation of cell motility, and tumor metastases *in vivo*. More recently, RANTES has been shown to enhance the migration of chondrosarcoma cells through increased MMP-3 production [40]. S100A4 might be implicated in this process as well, since previously it has been shown that S100A4 could trigger MMP activation in tumor cells and synovial fibroblasts and in endotheial cells [6], [ 7], [ and 41].

The precise mechanism of RANTES-mediated activation of S100A4 release remains to be solved. However, here we disclosed certain aspects of the cellular events triggered by RANTES, particularly associated with S100A4 secretion. The S100A4 protein lacks a secretion signaling peptide. As we have shown, consistent with others [42], S100A4 is not secreted by the classical ER/Golgi route. We explored which alternative mechanism may be responsible for S100A4 release. Secretion of S100A4 from fibroblasts does not involve lysosomes, which are not exclusively restricted to protein degradation but also have been shown to be involved in secretory process of different cell types [43].

We found that in response to RANTES both tumor and stroma cells stimulate the release of plasma membrane covered microparticles, also known as microvesicles. One of the cargo proteins of the microparticles as we found, is S100A4. The release of microparticles from various cell types is a well-known phenomenon [27], [ 28], [ 44], [ and 45]. These membrane vesicles are relatively large and heterogeneous, ranging in size from 70 nm and more than 1000 nm. Vesicle shedding resembling cell blebbing during apoptosis is an active process that occurs in response to different stimuli also in living cells showing no signs of cell death [44]. In our studies we carefully examined that the formation of microparticles, which occurs in cells in response to RANTES is not associated with apoptosis (Supplementary data, Fig. S1). Recently, the contribution of microparticles to vital biological processes has been documented, including the mediation of horizontal, cell-to-cell transfer of RNA and proteins such as receptors for growth factors and cytokines [27], [ 28], [ 44], [ and 45]. Extracellular microparticles released from cells circulate in tumor microenvironment but can also be transferred by bodily fluids and affect cells at distal sites as well [26], [45]. Interestingly, the pattern of interaction and internalization of microparticles depends significantly on the recipient cell type. Thus, in our cell models the attachment of S100A4-positive microparticles to the membrane and the uptake to the cytoplasm, was much more pronounced in fibroblasts (5MEF) compared to VMR tumor cells, which indicate that the horizontal microparticle transfer is an active process and requires acceptor molecules (e.g. receptors) on the cell surface. We also confirmed observations made by others [28], [45] on the functional significance of microparticles released into the extracellular space from various types of cells such as fibroblasts and macrophages. Additionally we disclosed the biological activity of micropraticle-carried S100A4 in stimulation of FN production and activation of fibroblasts migration. These effects are obviously less pronounced in response to S100A4-negative microparticles.

Importantly, we are the first demonstrating a pathway where RANTES and S100A4 cooperatively influence cell migration. Thus, S100A4 blockade by specific antibodies appeared to considerably decelerate RANTES-induced migration of S100A4-positive fibroblasts, directly confirming that RANTES-driven cell migration is mediated at least partially by S100A4. These data are consistent with the previously reported effects of S100A4 on cell motility and migration [13], [46].

Animal studies performed indicate that overexpression of RANTES in tumor cells confers more pronounced metastatic phenotype on tumor cells independently on S100A4. However a clear synergism between RANTES and S100A4 in metastasogenesis supports the significance of the RANTES/S100A4 interplay *in vivo*. Thus, overexpression of RANTES in two different cell lines (VMR and CSML0) notably increased the metastatic capacity of tumor cells and resulted in the ability of tumor cells to colonize much broader spectrum of organs such as ovaries, kidney, and lymph nodes, in addition to lung and liver. It could be associated with RANTES-mediated activation of the immune system, such as the enlargement of the lymph nodes and thymus and infiltration of organs with CD45 cells in both wild type and S100A4 knockout mice (unpublished observations). Notably this phenotype is much more pronounced in wild type (S100A4+/+) compared to S100A4-depleted animals. The most striking effect of RANTES/S100A4 interplay is observed in the metastatic burden in lungs. The impact of S100A4 on metastasis in organs other than the lung is not so apparent. Thus RANTES-overexpressing cells raise significantly more (82-fold) metastases in lung compared with control tumor cells in S100A4+/+ mice, whereas this difference is notably lower (5.6-fold) in the S100A4 (−/−) mice. Moreover, S100A4 emerging in the serum of mice injected with RANTES-VMR cells but not VMR-vector control cells substantiates our hypothesis regarding the role of the RANTES/S100A4 pathway in metastasis. Additionally, the role of S100A4 in immune cell attraction must be acknowledged because of our recent results showing a strong S100A4-directed attraction of T-lymphocytes in *in vitro* and *in vivo* models [47].

We hypothesize that the specificity of RANTES/S100A4 circuit in stimulation of lung metastases might be associated with the formation of specific pre-metastatic niches in the lungs [48]. An important role of signals transmitted from primary tumor cells (e.g. cytokines) in the stimulation of pro-inflammatory members of the S100 family (S100A8/S100A9) and FN followed by attraction of immune cells and their progenitors from bone marrow to the site of metastasis and the formation of “pre-metastatic” niches were recently reported [49], [50]. We may predict an essential role for the RANTES/S100A4 pathway in this process, in which RANTES, in cooperation with other factors (e.g. cytokines) stimulates S100A4 release resulting in attraction and activation of fibroblasts and immune cells such as T-cells [47], whose activity in tumors and at sites of “pre-metastatic” niches could facilitate tumor cell homing and proliferation in distal organs (e.g. lungs).

Although an ultimate role of RANTES as a tumor-derived factor in the promotion of a S100A4-associated pro-malignant phenotype is postulated here, determining the precise role of RANTES requires investigating the role of additional, as yet unidentified, co-factors (e.g. CSML0-CM). Other researchers also mentioned the need to identify co-factors for RANTES involved in its pro-metastatic effects [35]. Further investigations need to elucidate more details of signaling mechanisms and additional co-factors involved in the pro-metastatic RANTES/S100A4 circuit as well.

In summary, we have demonstrated that RANTES and S100A4 reciprocally stimulating each other participate in a pathway conferring more pronounced metastatic phenotype on tumor cells. The results presented here highlight certain aspects of the mechanism behind this pathway and open perspectives for its therapeutic targeting.

## Materials and Methods

### Ethics Statement

Animals were propagated in pathogen-free environment according to FELASA guidelines.

### Cell lines and recombinant proteins

Mouse macrophages and the monocyte RAW264.7 cell line were kindly provided by Dr. Svetlana Panina (Novo Nordisk, Denmark).VMR, CSML100, and CSML0 mouse adenocarcinoma cell lines were derived from two independent spontaneous tumors in A/Sn mice [51] and 4MEF/S100A4+/+ and 5MEF S100A4−/− mouse embryonic fibroblasts and were isolated and cultured as described previously [8].

For retrovirus-mediated gene transfer, mouse RANTES cDNA was synthesized using the forward RANTES-specific primer 5′-CGC GGG TAC CAT GAA GAT CTC T-3′ and reverse primer 5′-CCC TCT ATC CTA GCT CAT CTC C-3′ and cloned into a *pBabepuro* vector. Selection of the infected cells was performed according to a previous report [52].

An active oligomeric fraction of S100A4 was obtained from recombinant 6xHis-tagged protein by gel filtration as described previously [15]. Recombinant RANTES was purchased from AH Diagnostics (catalog no. PMC1055, Denmark).

### Protein analysis

Cells were grown to 90% confluency in T25, T75, or T125 flasks in Dulbecco's Modified Eagle Medium (DMEM) supplemented with 10% fetal calf serum (FCS). The medium was exchanged with fresh medium supplemented with the proteins of interest. The cultures were sustained for the required periods of time. For the analysis of secreted proteins, CM were harvested and filtered through a 0.45 µm membrane (Schleicher & Schuell, Germany). The adherent cells were trypsinized and counted in 0.25% Trypan blue. Only the experiments with not less than 99% of alive cells were processed further. Additionally, cell viability was checked for each cell culture using the LDH Cytotoxicity Detection Kit (Clontech) according to manufacturer's instructions.

RayBio Mouse Cytokine Antibody Array 3 and 4 were purchased from (RayBiotech, Inc) and the cytokine analyses in cell culture CM were performed according to the manufacturer's instructions. Western blot analysis and sandwich enzyme-linked immunosorbent assay (ELISA) were performed according to a previous report [16].

### Quantitative real-time polymerase chain reaction (qPCR)

Total cellular RNA was isolated according to a previous report [53]. First-strand cDNA synthesis was performed using SuperScript II RT (Invitrogen) with random primers according to the manufacturer's instructions. Real-time polymerase chain reaction (PCR) was performed using a LightCycler 2.0 instrument following the manufacturer's instructions (Roche Applied Science, USA) The following primers were used: mouse RANTES forward primer (5′-CATATGGCTCGGACACCA-3′), mouse Rantes reverse primer (5′-ACACACTTGGCGGTTCCT-3′), mouse S100A4 forward primer (5′-TTGTGTCCACCTTCCACA-3′), mouse S100A4 reverse primer (5′-GCTGTCCAAGTTGCTCAT-3′), glyceraldehyde 3-phosphate dehydrogenase (GAPDH) forward primer for normalization (5′- CCAGCAAGGACACTGAGCAA-3′), and GAPDH reverse primer (5′- GGGATGGAAATTGTGAGGGA-3′).

### Isolation of microparticles

Isolation of microparticles shedded from adherent cell cultures was performed as described previously [28], [29]. Twenty-four hours after incubation, CM were collected from cells at 75–90% confluence and subjected to centrifugation first at 500×*g* for 5 min, then at 1500×*g* for 20 min to remove floating cells and cellular debris. Microparticle fractions were obtained after centrifugation at 100,000×*g* for 2 h. Microparticle pellets were washed in large quantities of phosphate-buffered saline (PBS) and re-centrifuged at 100,000×*g* for 2 h. The pelleted microparticles were resuspended in DMEM or PBS depending on the following procedures. Microparticle protein content was determined using the Bradford assay (Bio-Rad).

### *In vitro* wound healing assay

The monolayer of MEF cells grown in DMEM (Gibco Invitrogen) was supplemented with 2% FCS in six-well plates up to 100% confluence. Two horizontal and two vertical scratches (wounds) were made in each well with a plastic loop. Cells were washed with fresh media, and images were taken as point 0. The media of interest were added, and wound healing was monitored by taking serial photos of the marked areas. The extent of wound healing was determined by measuring the width of a residual wound inside the marked area using Multi Gauge software (Fujifilm). For each treatment, three parallel wells were used with four marked areas each. The relative migration was calculated as the average width at different time-points.

### Animal experiments

Eight- to 13-week-old S100A4−/− knockout and wild type mice on an A/Sn genetic background were used in the two experiments. All animals were maintained according to the guidelines of the Federation of European Laboratory Animal Science Association for the care and use of laboratory animals. Animals received an intravenous lateral tail vein injection of 0.5×10^6^ tumor cells suspended in 0.1 ml PBS. Mice were examined and weighed every day. At the time of euthanasia, the visceral organs were removed by dissection, fixed in 4% formaldehyde, and paraffin-embedded. Metastasis was assessed in histological sections and correlated with the whole section space by taking overlapping light images of histological sections and assessing the metastasis/tissue area ratio using Multi Gauge software (Fujifilm). Metastatic burden was determined as a ratio of the total area covered by tumor cells to the total tissue area in the section.

### Immunofluorescence

Immunofluorescence was performed according to a previous report [9]. The antibodies used were rabbit anti-S100A4 (1∶4000), rabbit anti-fibronectin (1∶4000) (Biomedical Technologies, USA), and rat anti-LAMP1 (1∶5000) (Becton Dickinson, USA). Secondary Alexa Fluor antibodies were purchased from Molecular Probes, USA) and used in a dilution of 1∶1500. To visualize polymerized actin, TRITC-conjugated Phalloidin was used. Nuclei were stained with TO-PRO 3 iodide (1∶5000) (Molecular Probes, USA). Cover slips were mounted with Fluoromount-G (Southern Biotechnology, USA) and analyzed and quantified using a laser-scanning confocal LSM 510 microscope and software (Carl Zeiss, Inc).

Plasma membrane labeling of live adherent cells and isolated fraction of microparticles was done using Lipophilic Styryl dye FM**®** 1-43FX (Molecular Probes, USA) according a protocol recommended by the manufacturer.

### Statistical Analysis

All data were analyzed using Graph Pad Prism 5.0 statistical software (GraphPad Software Inc.). The differences between two groups were analyzed using Non-parametric Mann- Whitney test. Two sided p-values of <0.05 were regarded significant.

## Supporting Information

Figure S1 Analysis of CSML100 cells viability after 4 h of stimulations. As a positive control for apoptosis cells were treated with 1µM Staurosporine (STA). A. Cytostaining of fixed CSML100 cells with DAPI (4′,6-diamidino-2-phenylindole). B. Western blot analysis of Caspase-3 activation.
(0.42 MB TIF)

Figure S2 Western blot analysis of RANTES in cell lysates (CL) and condition media (CM) from VMR cells infected with pBabe-puro vector (VMR-mock) and pBabe-puro-RANTES (VMR-RANTES). Rabbit polyclonal anti-RANTES antibodies (Chemicon, USA, Cat.AB2109P) were used.
(0.05 MB TIF)
