# Supplementary figures and images for: Metastasis-Inducing S100A4 and RANTES Cooperate in Promoting Tumor Progression in Mice
Source: PLoS One. 2010 Apr 28;5(4):e10374. doi: 10.1371/journal.pone.0010374 (PMC2860983; doi:10.1371/journal.pone.0010374)

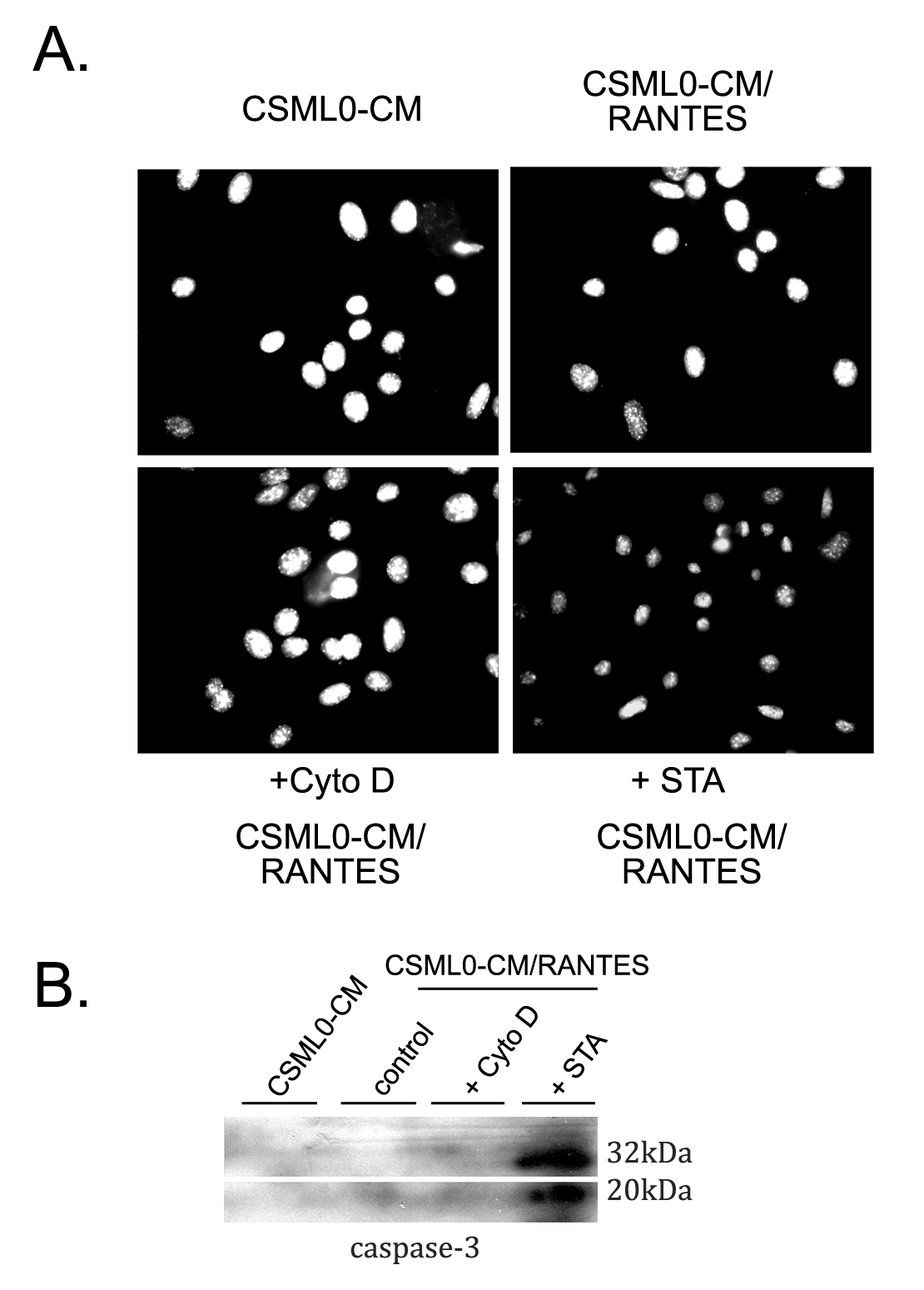

Supplement: Figure S1 — Analysis of CSML100 cells viability after 4 h of stimulations. As a positive control for apoptosis cells were treated with 1µM Staurosporine (STA). A. Cytostaining of fixed CSML100 cells with DAPI (4′,6-diamidino-2-phenylindole). B. Western blot analysis of Caspase-3 activation. (0.42 MB TIF) [file pone.0010374.s001.tif]

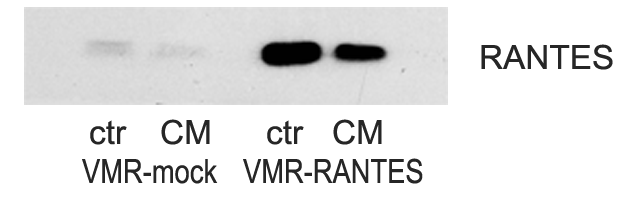

Supplement: Figure S2 — Western blot analysis of RANTES in cell lysates (CL) and condition media (CM) from VMR cells infected with pBabe-puro vector (VMR-mock) and pBabe-puro-RANTES (VMR-RANTES). Rabbit polyclonal anti-RANTES antibodies (Chemicon, USA, Cat.AB2109P) were used. (0.05 MB TIF) [file pone.0010374.s002.tif]
